# Ze-Qi-Tang Formula Induces Granulocytic Myeloid-Derived Suppressor Cell Apoptosis via STAT3/S100A9/Bcl-2/Caspase-3 Signaling to Prolong the Survival of Mice with Orthotopic Lung Cancer

**DOI:** 10.1155/2021/8856326

**Published:** 2021-04-01

**Authors:** Zi-hang Xu, Yang-zhuangzhuang Zhu, Lin Su, Xue-yang Tang, Chao Yao, Xiao-ning Jiao, Yi-fei Hou, Xiao Chen, Lu-yao Wei, Wan-tao Wang, Jie Wang, Chen-yuan Gong, Xian-dan Zhu, Fei Zhang, Shi-guo Zhu, Chun-pu Zou

**Affiliations:** ^1^School of Basic Medical Science, Shanghai University of Traditional Chinese Medicine, Shanghai 201203, China; ^2^School of Chinese Medicine, Hunan University of Chinese Medicine, Changsha 410208, China; ^3^Center of Technology Experiment, Shanghai University of Traditional Chinese Medicine, Shanghai 201203, China; ^4^Department of General Surgery, Xinhua Hospital, Affiliated to Shanghai Jiao Tong University, School of Medicine, Shanghai 200092, China

## Abstract

Non-small-cell lung cancer (NSCLC) remains the most common malignancy with the highest morbidity and mortality worldwide. In our previous study, we found that a classic traditional Chinese medicine (TCM) formula Ze-Qi-Tang (ZQT), which has been used in the treatment of respiratory diseases for thousands of years, could directly inhibit the growth of human NSCLC cells via the p53 signaling pathway. In this study, we explored the immunomodulatory functions of ZQT. We found that ZQT significantly prolonged the survival of orthotopic lung cancer model mice by modulating the tumor microenvironment (TME). ZQT remarkably reduced the number of MDSCs (especially G-MDSCs) and inhibited their immunosuppressive activity by inducing apoptosis in these cells via the STAT3/S100A9/Bcl-2/caspase-3 signaling pathway. When G-MDSCs were depleted, the survival promotion effect of ZQT and its inhibitory effect on lung luminescence signal disappeared in tumor-bearing mice. This is the first study to illustrate the immunomodulatory effect of ZQT in NSCLC and the underlying molecular mechanism.

## 1. Introduction

Lung cancer is a serious malignant lung disease, and is the leading cause of cancer death in men and women worldwide [1]. Non-small-cell lung cancer (NSCLC) accounts for 85-90% of all lung cancers [2]. The 5-year survival rate of early-stage NSCLC patients who received surgical excision of the tumor is 36%-83%, depending on the stage of the disease [3]. Current treatment for NSCLC is mainly a comprehensive regimen of chemoradiotherapy, targeted therapy, and immunotherapy. However, these interventions often have strong side effects [4–6], and after the treatment, patients are still at risk of relapse and drug resistance. Therefore, novel therapeutic approaches that can be used to inhibit NSCLC and prevent relapse and drug resistance are urgently needed. Traditional Chinese medicine (TCM), as a complementary and alternative medical treatment, also plays an important role in the treatment of tumor diseases. At present, accumulating researches indicate that TCM therapy can obviously improve the quality of life of NSCLC patients, significantly prolong their survival, and apparently alleviate the clinical symptoms of the patients. In addition, TCM is less toxic and has fewer side effects [7, 8]. Therefore, TCM, as a popular and promising anticancer agent, has been gaining more and more attention.

The tumor microenvironment (TME) is the cellular environment of a tumor, which not only plays a pivotal role in tumor initiation, progression, and metastasis but also has profound effects on therapeutic efficacy [9]. Studies have shown that the antitumor effect of TCM mainly depends on the ability of TCM to modulate TME [10–12]. Myeloid-derived suppressor cells (MDSCs) are an important component of TME. Their function is to inhibit T lymphocytes and natural killer- (NK-) cell-mediated anticancer immunity, and to recruit T regulatory cells at the same time to further enhance the immunosuppressive effect to promote the progress of lung cancer [13]. According to the heterogeneity of MDSCs in morphology, MDSCs can be divided into granulocytic MDSCs (G-MDSCs/PMN-MDSCs) and monocytic MDSCs (M-MDSCs), of which G-MDSCs are the prevalent population of MDSCs. Murine G-MDSCs are defined as CD11b^+^Gr-1^+^Ly6G^+^Ly6C^lo^, while M-MDSCs are CD11b^+^Gr-1^+^Ly6G^−^Ly6C^hi^ [14, 15]. Ze-Qi-Tang (ZQT), a widely used TCM formula in China, was initially documented in the “Jin Gui Yao Lue” of the Eastern Han Dynasty. Based on the properties of ZQT, the main clinical application of ZQT is to treat pulmonary diseases, such as cough, asthma, chest pain, hydrothorax, and lung cancer [16, 17]. In our previous study, we found that ZQT induced mitochondria-mediated apoptosis in human NSCLC cells (H460 and A549) via upregulation of p53 and downregulation of Cyclin B1 and Cdk2 [18]. This is the direct cell growth inhibitory effect of ZQT on H460 and A549 cells. However, the immunoregulatory effect of ZQT on TME of an orthotopic NSCLC mouse model has not been fully investigated yet. In this study, we found that ZQT treatment significantly prolonged the survival time of orthotopic NSCLC-bearing mice. We also found that ZQT treatment changed the profile of immune cells in TME: MDSCs (especially G-MDSCs) were significantly decreased due to apoptosis, which led to a remarkably increased population of CD8^+^ T cells. To our best knowledge, this is the first study to elucidate the mechanism through which ZQT relieves the immunosuppressive effect of G-MDSCs on CD8^+^ T cells. On one hand, ZQT induces apoptosis in G-MDSCs; on the other hand, it also inhibited the immunosuppressive activity of G-MDSCs in TME, which demonstrates the multitarget cancer suppressive effect of ZQT.

## 2. Materials and Methods

### 2.1. Preparation of ZQT

All crude drugs (as shown in Supplementary Table 1) were purchased from Shanghai Kangqiao Chinese Medicine Pieces Company. Botanical identification was authenticated by Professor Xiao Chen (Shanghai University of Traditional Chinese Medicine, China). ZQT was first recorded in the Chinese ancient book “Jin Gui Yao Lue.” Preparation of the lyophilized ZQT powder was made as described previously [19]. Briefly, 495 g herbal mixture (in a 30 g mixture, the weight ratio is as follows: 15 : 10 : 10 : 10 : 6 : 6 : 6 : 6) was extracted three times with boiling water and further evaporated into 0.144 g/mL extracts before being lyophilized into powder. The quality control analysis of lyophilized ZQT powder using HPLC followed the method described in a previous publication [18].

### 2.2. Reagents

The following reagents were used: Matrigel basement membrane matrix (Corning, CAT#356234); Hygromycin B (MK Bio, CAT#MS0003-1G); FBS (fetal bovine serum), PS (penicillin-streptomycin), DMEM (Dulbecco's modified Eagle's medium), Trypsin-EDTA (0.25%) (Thermo Fisher Scientific, CAT#10091148, 15140122, 11995065, and 25200072); XenoLight D-Luciferin-K^+^ Salt Bioluminescent Substrate (PerkinElmer, CAT#122799); Brilliant Violet 510™ anti-mouse CD335 (NKp46), PE/Dazzle™ 594 anti-mouse CD11c, Brilliant Violet 650™ anti-mouse I-A/I-E, Brilliant Violet 421™ anti-mouse F4/80, Alexa Fluor® 700 anti-mouse CD4, Brilliant Violet 605™ anti-mouse CD8a, APC anti-mouse CD8, FITC anti-mouse CD4, PE anti-mouse CD3, FITC anti-mouse CD11b, PE anti-mouse Gr1, APC anti-mouse Ly6G, PerCP5.5 anti-mouse Ly6C, purified anti-mouse CD28, PE-Cy7 anti-mouse CD107*α* (LAMP-1) (BioLegend, CAT#137623, 117347, 107641, 123137, 100536, 100743, 100712, 100406, 100205, 101206, 108407, 127614, 128011, 102101, and 121619); Red Cell Lysis (Biosharp, CAT#BL503B); CFSE (Sigma-Aldrich, CAT#21888); EasySep Mouse T Cell Isolation Kit, EasySep Mouse MDSC Isolation Kit (STEMCELL, CAT#19851 and 19867); InVivoMab anti-mouse Ly6G (1A8), InVivoMab rat IgG2a isotype control (Bio X Cell, CAT#BE0075-1 and BE0089); Mouse IFN-*γ* ELISA Set (BD Biosciences, CAT#555138); STAT1, p-STAT1, STAT3, p-STAT3, S100A9, cl-PARP, Arg-1, Ly6G, Cytochrome c, Cyclin D1 (Cell Signaling Technology, CAT#14994, 58D6, 9139T, 4113S, 73425, 9548S, 93668, 87048, 11940T, and 55506T); 1x TBS (Tris-buffered saline) buffer, Tween 20 (Sangon Biotech, CAT#C508113 and A600560); Immobilon Western Chemiluminescent HRP Substrate (Millipore, CAT# WBKLS0500); rabbit anti-Ki67 antibody (Abcam, CAT#ab16667); anti-Bcl-2 (rabbit) antibody (Rockland Immunochemicals, CAT# 200-401-Z43); purified anti-Bax (Biolegend, CAT#633601); cl-caspase-3 (StressMarq Biosciences Inc., CAT# SPC-1319); DeadEnd Fluorometric TUNEL System (Promega, CAT#REF: G3250); Reactive Oxygen Species Assay Kit (Beyotime, CAT#S0033S), Cell Cycle and Apoptosis Analysis Kit (Beyotime, CAT#S0033S and C1052).

### 2.3. Cell Lines and Cell Cultures

The NSCLC cell line LLC (Lewis lung cancer), derived from C57BL/6 mice, was purchased from the Cell Bank of the Chinese Academy of Sciences. LLC-Luc cells that stably expressed luciferase were conserved in our own laboratory. The cells were maintained in DMEM medium supplemented with 1% penicillin-streptomycin and 10% fetal bovine serum (FBS). Cells were cultured in a cell incubator with 5% CO_2_ at 37°C.

### 2.4. In Vivo Treatments

The experiments on the animals were approved by the Institutional Animal Care and Use Committee of Shanghai University of Traditional Chinese Medicine in accordance with the ethical guidelines of the National Institutes of Health Guide for the Care and Use of Laboratory Animals. C57BL/6 mice aged 6-8 weeks (male) and weighing 20 ± 2 g were purchased from Shanghai SLAC Laboratory Animal Co., Ltd., and maintained in a specific pathogen-free environment. Mice were fed a basal diet at conditions of22 ± 1°Ctemperature, 12 h dark/light cycle, and 60% relative humidity. The orthotopic lung cancer mouse model was established as described previously. To be brief, 2.4 × 10^6^ cells/mL LLC-Luc cells suspended in PBS were mixed with Matrigel (at a 1 : 1 volume ratio) before being aspirated into insulin syringes (100 *μ*L/mouse). After anesthesia, mice were placed in a prostrate position on a sterile surgical station. An approximately 1 cm skin incision was made on the left chest, and the cell suspension was directly injected into the lung. All 20 mice were randomly divided into the following four groups (five mice per group): the control group and the Ze-Qi-Tang group of high, medium, and low doses (equal to 0. 342 g/mL, 0.171 g/mL, and 0.086 g/mL ZQT lyophilized powder according to the clinical dose as well as the description in the Chinese ancient book “Jin Gui Yao Lue”). On the same day of surgery, mice were intragastrically administered with a daily dose of either 200 *μ*L Ze-Qi-Tang or an equal volume of saline. The appearance of mice (mental state, locomotor activity, hair grooming, etc.) was observed and their survival was recorded. In vivo imaging was taken on days 4, 11, 18, and 28 after surgery to monitor tumor growth in mice. In another experiment, 20 model mice were intragastrically administered with a daily dose of 200 *μ*L Ze-Qi-Tang, intraperitoneally injected with 5 mg/mL STAT3 inhibitor (S3I-201, CSNpharm), separately or combined, 3 times a week for 4 weeks. Normal saline was used as control.

### 2.5. H&E Staining

Tumors were fixed in 4% paraformaldehyde for 12 h, embedded in paraffin, and cut into 4 *μ*m sections. The sections were then processed for hematoxylin and eosin staining. The sections stained with H&E were scanned and observed under a microscope.

### 2.6. Tissue Processing and Flow Cytometry

All tissues were used freshly. After the mice were anesthetized with 1.5% sodium pentobarbital, whole blood was taken from the eyeball. Spleen, bone marrow, and tumor tissues from the lungs were processed by mechanical dissociation followed by RBC lysis. Suspensions were resuspended with PBS prior to cell surface staining with an appropriate amount of fluorochrome-conjugated antibodies. Data were acquired and analyzed on a CytoFLEX using a CytExpert software (Beckman Coulter).

### 2.7. TUNEL Assay

The apoptosis of MDSCs in tumor was detected using the DeadEnd™ Fluorometric TUNEL System.

### 2.8. Immunohistochemistry (IHC)

Tumors were fixed in 4% paraformaldehyde for 12 h, embedded in paraffin, and cut into 4 *μ*m sections. Then, these sections were deparaffinized and rehydrated with a series of gradient concentrations of alcohol, and incubated with 3% hydrogen peroxide to block the activity of endogenous peroxidase. Antigen retrieval was achieved by heating the tissue block in 0.01 mol/L sodium citrate buffer and washed with PBS. Nonspecific binding was blocked with 5% BSA. Each section was then incubated with antibodies against S100A9 (1 : 800), Ly6G (1 : 200), Ly6C (1 : 200), CD4 (1 : 200), and CD8 (1 : 800), respectively, followed by incubation with the HRP-labeled secondary antibody. Finally, the sections were subjected to staining with a diaminobenzidine substrate-chromogen solution, followed by staining with hematoxylin.

### 2.9. Immunofluorescence (IF) Staining

Purified MDSCs or G-MDSCs were resuspended in PBS, fixed in 4% paraformaldehyde for 30 min, and washed twice with PBS. Cells were treated with 1% Triton X-100 for 10 min, washed twice with PBS, and incubated with Ki67 (1 : 200), STAT3 (1 : 1,000), and cl-caspase-3 (1 : 1,000) antibodies, respectively. Next, cells were incubated with the fluorescence-conjugated secondary antibody in the dark. Finally, the cells were stained with DAPI and examined under the fluorescence microscope.

### 2.10. RT-qPCR

RNA was extracted from tumor using the Tissue RNA Purification Kit PLUS. cDNA was synthesized using the 4 × Reverse Transcription Master Mix Kit. The following primers were used: Arg-1 (forward 5′-CTCCAAGCCAAAGTCCTTAGAG-3′; reverse 5′-AGGAGCTGTCATTAGGGACATC-3′); iNOS (forward 5′-CTGTGTG CCTGGAGGTTCTG-3′; reverse 5′-CCAATCTCTGCCTATCCGTCTC-3′); S100A9 (forward 5′-TGGCCAACAAAGCACCTTCT-3′; reverse 5′-TGTGTCCAGGTCCTC CATGA-3′); GZMB (forward 5′-TCGACCCTACATGGCCTTAC-3′; reverse 5′-TGGGGAATGCATTTTACCAT-3′); PRF1 (forward 5′-GATGTGAACCCTAG GCCAGA-3′; reverse 5′-GGTTTTTGTACCAGGCGAAA-3′); and *β*-actin (forward 5′-AGAGGGAAA TCGTGCGTGAC-3′; reverse 5′-CAATAGTGACCTGGCCG T-3′). Gene expression was determined relative to *β*-actin using the indicated primers on a QuantStudio 3 (Thermo Fisher Scientific) PCR instrument using the 2 × SYBR Green qPCR Master Mix Kit.

### 2.11. ELISA

The level of IFN-*γ* in blood was determined using the commercially available Mouse IFN-*γ* ELISA Set following manufacturer's protocol. Optical density at a wavelength of 450 nm was determined using a microtiter plate reader.

### 2.12. Reactive Oxygen Species (ROS) Staining

ROS production in tumor was detected using a SpectraMax190 Microplate Reader following the manufacturer's protocol.

### 2.13. Western Blotting

Tumor cell lysates were obtained by lysing the tumor cells in RIPA lysis buffer mixed with a phosphatase inhibitor and a protease inhibitor (Beyotime, China), which was heated at 99°C for 15 min. An equal amount of protein (40 *μ*g) was separated by SDS-PAGE on an 8-12% acrylamide gel for electrophoresis and transferred to a polyvinylidene difluoride (PVDF) membrane (Millipore). Primary antibodies were diluted in skimmed milk powder prepared from TBST: rabbit monoclonal anti-STAT3 antibody, 1 : 1000 (Cell Signaling Technology, USA); rabbit monoclonal anti-pSTAT3 antibody, 1 : 1000 (Cell Signaling Technology, USA); mouse monoclonal anti-Arg-1 antibody, 1 : 500 (Santa Cruz Biotechnology, USA); Cyclin D1 (E3P5S) XP® Rabbit mAb, 1 : 1000 (Cell Signaling Technology, USA); cytochrome c (D18C7) Rabbit mAb, 1 : 1000 (Cell Signaling Technology, USA); mouse monoclonal anti-beta-actin, 1 : 5000 (Proteintech, China). Each blot was incubated with the Chemiluminescent HRP Antibody Detection Reagent (Millipore, USA) and imaged using Image Lab software (Bio-Rad, USA).

### 2.14. MDSC Depletion In Vivo

For the depletion of MDSCs, mice were treated daily with an anti-mouse Ly6G antibody or control rat IgG (Bio X Cell, USA) via intraperitoneal injections for 14 days before surgery. Afterward, the model mice were given maintenance treatment every other day.

### 2.15. Cell Sorting

Tumor cell suspensions were sorted on a EasySep™ magnet (STEMCELL Technologies Inc., USA) using the MojoSort Mouse Isolation Kit (STEMCELL Technologies Inc., USA) to select T-lymphocytes, CD8^+^ T cells or MDSCs according to the manufacturer's protocol. Briefly, non-CD3^+^ T cells or non-MDSCs were depleted by incubating a tumor cell suspension with the biotin antibody cocktail, followed by incubation with magnetic Streptavidin Nanobeads. In other experiments, tumor cell suspensions were stained with CD11b and Gr-1 antibodies and incubated in the dark for 30 min. G-MDSCs were isolated using flow cytometry. The purity of cells enriched from tumor populations was consistently >90% as assessed by flow cytometry.

### 2.16. T-Lymphocyte Proliferation Assay

CD3^+^ T cells isolated from tumor were stained with 5 *μ*M carboxyfluorescein succinimidyl ester (CFSE). Afterward, MDSCs isolated from ZQT-treated tumor-bearing mice (Z-MDSCs) or from control-treated mice (C-MDSCs) as effector cells were incubated with purified T cells as well as CD3 and CD28 antibodies at E : T, 0 : 1, 1 : 1, 1 : 2, and 1 : 4, respectively, for 72 hours. APC anti-mouse CD8 antibody was added before detection and incubated for 30 min. Flow cytometry was used to quantify 72 h of CFSE dilution. Proliferation of the cells was quantified as the average number of divisions for all cells in the culture (division index) using FlowJo software.

### 2.17. T-Lymphocyte Degranulation Assay

The LLC cell line and CD3^+^ T cells isolated from the tumor of mice in all groups were adjusted to 1 × 10^6^ cells/mL and cocultured at E : T, 100 : 1. The CD107*α* antibody and its isotype control were added and incubated for 5 h at 37°C and 5% CO_2_. Then, CD3 and CD8 antibodies were added before being incubated for 30 min in the dark. The amount of degranulated cells was quantified using FlowJo software. Data were acquired and analyzed on CytoFLEX using a CytoExpert software (Beckman Coulter).

### 2.18. Cell Apoptosis and Cell Circle Assay

CD3^+^ T cells or MDSCs isolated from tumor were resuspended with binding buffer. Annexin V-FITC and PI were added in CD3^+^ T cells or MDSCs before being incubated on ice for 30 min in the dark. For cell cycle analyses, G-MDSCs were stained by propidium iodide (PI) under the manufacturer's protocol. The results were quantified using FlowJo software.

### 2.19. Statistical Analysis

All results were analyzed in GraphPad Prism Version 7.0. The survival curve was assessed using the log-rank (Mantel-Cox) test. Multiple comparisons were performed using one-way ANOVA with Tukey's comparison test. Statistical significance was indicated as follows: n/s = nonstatistical significance; ^∗^*p* < 0.05; ^∗∗^*p* < 0.01; and ^∗∗∗^*p* < 0.001.

## 3. Results

### 3.1. The Orthotopic Mouse Model of Lung Cancer Was Successfully Established by Intrapulmonary Injection of Lung Cancer Cells

**Figure 1:**
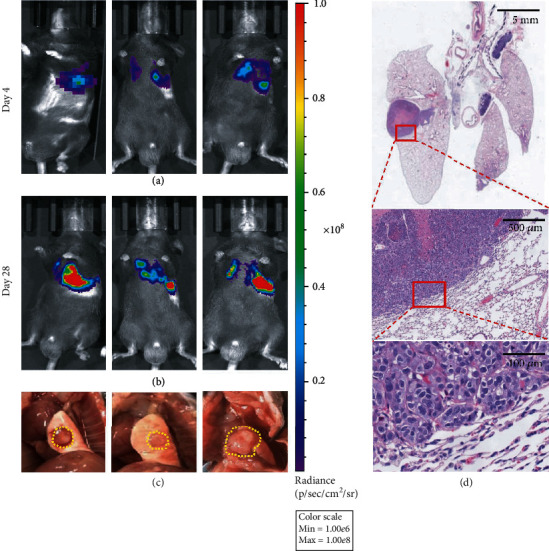
Establishment of the orthotopic lung cancer mouse model. The LLC-Luc cells (1.2 × 10^5^ cells/mouse) were orthotopic inoculated in the left lung of C57BL/6 mice. (a, b) The mice bioluminescence images on the 4th day and the 28th day after the tumor cell injection. (c) Harvested lung tissues. (d) H&E staining of lung tissues.

There is a theory in TCM which states that TCM treatment is “according to the etiology of the disease, using the corresponding drugs.” In other words, since Chinese herbal formula ZQT is used to treat pulmonary diseases in clinics, we should aim at the orthotopic tumor of the lung, rather than the subcutaneous tumor to explore its antilung cancer effect, which emphasizes the importance of the disease site. Therefore, we first generated an orthotopic lung cancer mouse model to imitate the clinical situation, instead of subcutaneous inoculation. Since using chemicals to induce a spontaneous lung cancer mouse model is time consuming, we compared in situ modeling methods such as intrapulmonary injection, bronchial infusion, thoracic injection, and tail vein injection. Luciferase-labeled Lewis lung cancer (LLC-Luc) cells and Matrigel suspended at a 1 : 1 ratio and inoculated directly into the lungs of mice (Figure S1) can efficiently generate lung nodules in mice with less time, low mortality, and fewer tumor cells, and can also be sensitively detected using live imaging. We intrapulmonarily inoculated 1.2 × 10^5^ cells in each C57BL/6 mouse. The mice rested for three days to recover from the surgery. On the fourth day after tumor cell inoculation, we started to take optical images of the mice. We found that a weaker fluorescent signal appeared in the mouse chest on the fourth day, while a stronger signal was detected on the 28th day after surgery, indicating that the signal was enhanced in a time-dependent fashion (Figures 1(a) and 1(b)). We euthanized mice after live imaging on day 28 and immediately harvested the mouse lung tissues. We found that there were visible nodules in the lungs, which corresponded to the luminescence signal in the mouse chest (Figure 1(c)). Besides, H&E staining of these lung tissues was performed, and the results indicated that the lung nodules we saw anatomically were histologically confirmed to be neoplasms (Figure 1(d)). The above data demonstrated the feasibility of the orthotopic lung cancer model established by intrapulmonary injection of luminescence-labeled cancer cells and provided a basis for our subsequent investigation.

### 3.2. The Medium Dose of ZQT Possessed the Most Potent Antitumor Capability

**Figure 2:**
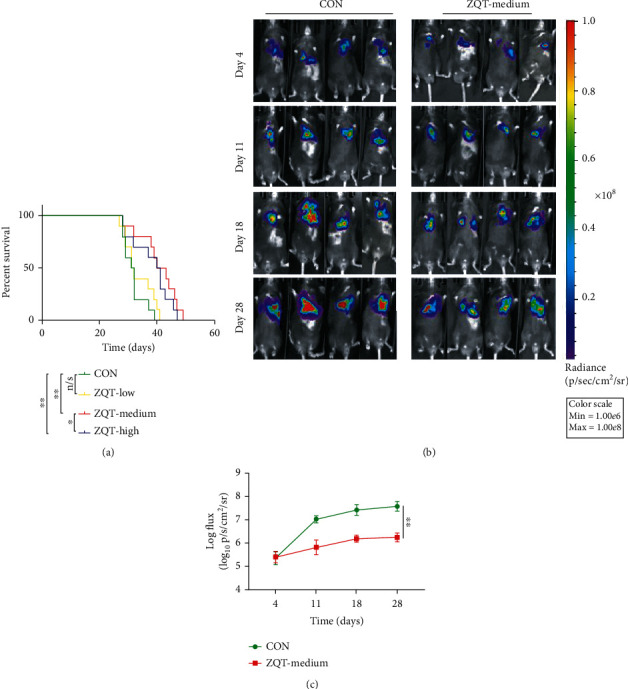
ZQT-medium group prolonged the survival of lung tumor-bearing mice. The C57BL/6 mice were intragastrically administered with normal saline or different doses of ZQT for 28 consecutive days after the orthotopic lung cancer mouse model was established. (a) Survival curves. (b, c) Live images taken on days 4, 11, 18, and 28, and the bioluminescence signal was recorded. *n* = 5. Data are expressed as the mean ± SD. n/s: nonstatistical significance. ^∗∗^*p* < 0.01; ^∗∗∗^*p* < 0.001.

Based on our previous work, we determined the dosage of the lyophilized powder of ZQT for mice according to the proportion of human consumption, and set three dose groups: high, medium, and low. Oral administration was initiated on the 4th day after the surgery of mice at a dose of 200 *μ*L per mouse, once a day, for 28 consecutive days. We first observed the effect of ZQT on the survival of tumor-bearing mice and found that the medium-dose and high-dose groups of ZQT can significantly prolong mice survival, especially in the medium-dose group (Figure 2(a)). Live imaging was also applied on the 4th day after surgery once a week for 4 weeks. Based on the living image data, the luminescence signal was remarkably inhibited in mice lungs in the ZQT medium-dose group compared with that of the saline control group, which supported our previous results (Figures 2(b) and 2(c)). These findings implied that, among the three doses, it was the ZQT medium-dose treatment that prolonged the survival of lung tumor-bearing mice. Therefore, we chose this dose for all succeeding experiments.

### 3.3. ZQT Reshaped the Immunogenic Tumor Microenvironment by Eliminating MDSCs and Enriching Antitumor T Cells

**Figure 3:**
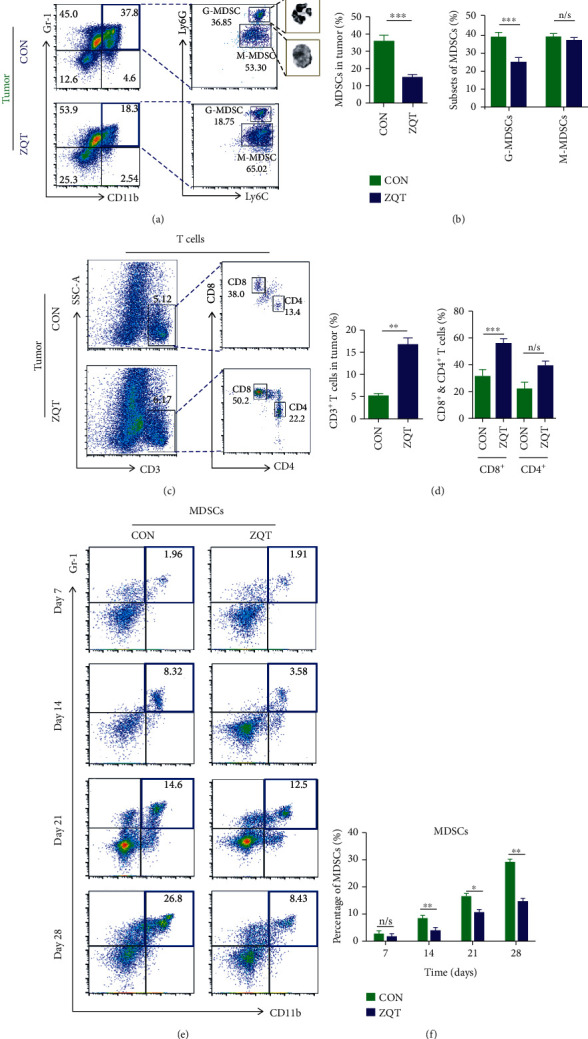
ZQT reduced the population of MDSCs but increased the population of CD8^+^ T cells in tumor tissue. Cells from the tumor tissue of tumor-bearing mice treated with normal saline or ZQT were analyzed by flow cytometry after staining with CD11b, Gr-1, Ly6G, Ly6C, CD3, CD4, and CD8 antibodies. (a–d) Percentages of MDSCs and T cells in tumor. (e, f) Percentages of MDSCs in tumor on days 7, 14, 21, and 28 after inoculation. *n* = 7. Data are expressed as the mean ± SD. n/s: nonstatistical significance. ^∗^*p* < 0.05; ^∗∗^*p* < 0.01; ^∗∗∗^*p* < 0.001.

Next, we further explored the role of ZQT in suppressing tumor growth in orthotopic lung tumor-bearing mice. Although our previous studies have shown that ZQT can directly inhibit the growth of human NSCLC cells H460 and A549 through the p53 signaling pathway, the TCM formula usually possesses multitarget tumor suppressive effects, and a large number of studies have shown that these formulas have potent immunoregulatory capability in TME. We reasoned that ZQT may also have this regulatory function to reshape TME. Therefore, we investigated whether the profiles of immune cells in TME were altered following ZQT treatment. A variety of immune cells that are closely associated with the reshaping of the TME were detected by flow cytometry, including MDSCs, NKs, Tregs, tumor-associated macrophages (TAMs), dendritic cells (DCs), CD4^+^ T cells, and CD8^+^ T cells (Figure S2). Among these cells, MDSCs (especially G-MDSCs) were significantly eliminated, while T cells (mainly CD8^+^ T cells) were enriched in lung tumor sites, which was consistent with the spleen data (Figures 3(a)–3(d)and Figures S3A–S3D). It is well known that MDSCs exert strong immunosuppressive activity in the TME, which is usually manifested as the suppression of the immune response of T cells, and MDSCs are recruited in a large number in the TME, contributing to the progress of cancer [20]. Therefore, we hypothesized that ZQT may play an antitumor role mainly by downregulating MDSCs in this orthotopic lung cancer mouse model. We also observed that the efficiency of ZQT in tumor-bearing mice showed a significant time-dependent trend, indicating that the MDSC inhibitory effect of ZQT is getting stronger with the increased action time of ZQT (Figures 3(e)–3(f)). Collectively, these data demonstrated that ZQT treatment was able to significantly reduce MDSC recruitment in TME and enhance T cell infiltration as well, through which the tumor-inhibitory immune microenvironment in an orthotopic lung cancer model was reshaped.

### 3.4. ZQT Attenuated the Immunosuppressive Effects of MDSCs on T Cells in the Tumor Microenvironment

**Figure 4:**
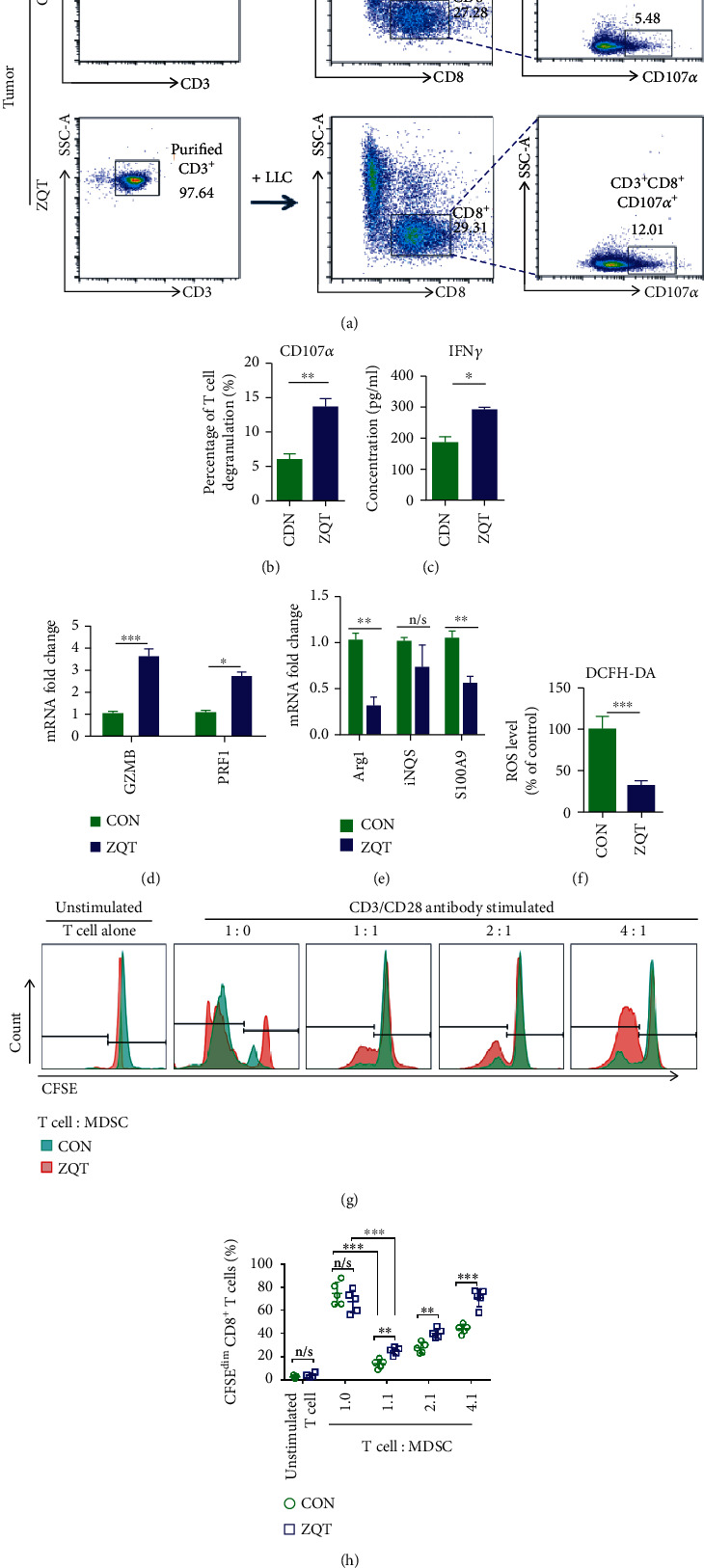
ZQT attenuated the immunosuppressive effect of MDSCs on T cells in the tumor microenvironment. (a, b) CD3^+^ T cells isolated from the tumor tissue of tumor-bearing C57BL/6 mice treated with normal saline or ZQT were cocultured with LLC cells for 4 h before incubating with CD3, CD8, and CD107*α* antibodies. Events shown were finally gated on CD3 and CD8. (c) ELISA analysis for the expression of IFN-*γ* in cells from the tumor tissue. (d, e) RT-qPCR quantitative analysis of Granzyme B, Perforin 1, Arg-1, iNOS, and S100A9 gene expression. (f) ROS assay for ROS production in tumor tissue from ZQT-treated mice or normal saline-treated mice. (g, h) T-lymphocyte in the spleen of wild-type C57BL/6 mice and MDSCs in tumor tissue of tumor-bearing mice treated with normal saline or ZQT were isolated using the MojoSort Mouse Isolation Kit. T-lymphocyte proliferation assay was used to show dose-dependent suppression of T cell proliferation by MDSCs isolated from tumor tissue of tumor-bearing mice treated with normal saline or with ZQT. CD3^+^ T cells isolated from tumor tissue stained with 5 *μ*M CFSE were incubated with MDSCs for 72 h. Meanwhile, CD3 and CD28 antibodies were added for CD3^+^ T cell stimulation. Flow cytometry analysis was performed to quantify 72 h CFSE dilution. *n* = 7. Data are expressed as the mean ± SD. n/s: nonstatistical significance. ^∗^*p* < 0.05; ^∗∗^*p* < 0.01; ^∗∗∗^*p* < 0.001.

We further explored the changes of immune function of MDSCs and T cells in orthotopic lung cancer mice after ZQT treatment. According to the literature, CD107*α* is a sensitive marker for the degranulation of T cells, which is directly related to the cytotoxic activity and can reflect the cytotoxic killing activity of T cells [21]. Therefore, after CD3^+^ T cells were sorted from the tumor of the model mice with magnetic beads (CD3^+^ > 90%), the expression of CD107*α* was detected by flow cytometry after coincubating with LLC cells for 4 h. The results showed that the content of CD107*α* was significantly upregulated, indicating that the degranulation ability and cytotoxic activity of T cells were greatly enhanced after tumor-bearing mice received ZQT treatment (Figures 4(a) and 4(b)). In addition, another cytokine IFN-*γ* which is related to T cell immune activity, and functional proteins perforin and granzyme that are associated with the cell-killing ability of T cells [22], were also determined by ELISA and RT-qPCR assay, respectively (Figures 4(c) and 4(d)). We found that the above biological markers that are associated with T cell activity were all significantly increased, further supporting the previous conclusion. Next, we detected the immunosuppressive activity markers of MDSCs, including Arg-1 (Arginase 1), iNOS (inducible nitric oxide synthase), S100A9 (S100 calcium-binding protein A9), and ROS (reactive oxygen species). The mRNA quantitative analysis results showed that the levels of Arg-1 and S100A9 were significantly downregulated, while the level of iNOS was not significantly altered post-ZQT gavage (Figure 4(e)). In addition, the ROS assay result demonstrated that the expression level of ROS was remarkably reduced in the ZQT treatment group compared with that in the saline control group (Figure 4(f)). T cells were hypersensitive to the immunosuppressive activity of MDSCs in TME which promote immune escape of tumor cells. Hence, we also evaluated the T cell proliferation suppression activity of MDSCs using T-lymphocyte proliferation assay. We sorted CD3^+^ T cells from wild-type mice according to a previous method, and we stained the cells with CFSE. Subsequently, MDSCs isolated from ZQT-treated tumor-bearing mice (Z-MDSCs) or from control-treated mice (C-MDSCs) were incubated with purified T cells that were stimulated using CD3 and CD28 antibodies for 72 h. Flow cytometry analysis was performed to detect the expression of CFSE which represent living cells. The results showed that the population of CD8^+^ T cells was significantly inhibited by C-MDSCs, while Z-MDSCs indicated much less capability for T cell inhibition in the T-lymphocyte proliferation assay (Figures 4(g)–4(h)). Taken together, the data demonstrated that the immunosuppressive effect of MDSCs on T cells was greatly compromised by ZQT treatment.

### 3.5. ZQT Induced Intensive Apoptosis of MDSCs to Reduce Its Proportion in TME

**Figure 5:**
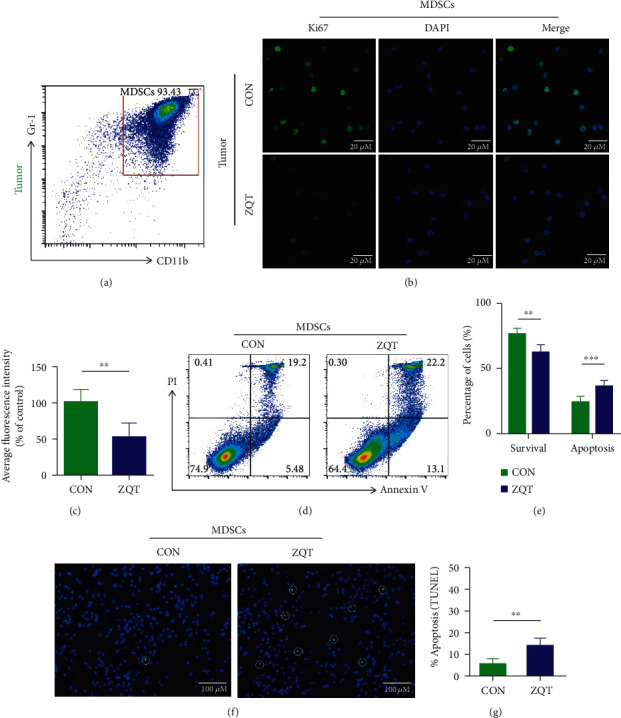
ZQT reduced MDSC population in TME by inducing intensive apoptosis in MDSCs. (a) MDSCs (CD11b^+^ Gr-1^+^) were isolated from tumor tissue using the MojoSort Mouse Isolation Kit, and their purity was determined using flow cytometry analysis. (b, c) The proliferation of MDSCs isolated from ZQT or normal saline-treated tumor-bearing mice were detected by IF staining of Ki67 (scale bar: 20 *μ*M). (d) Representative apoptosis analysis of MDSCs isolated from tumor tissue by flow cytometry stained with Annexin V and PI. (e) Percentage of survival and apoptotic MDSCs isolated from normal saline or ZQT-treated mice. (f, g) TUNEL staining of MDSCs collected from tumor tissue (original magnification: 200x). *n* = 5. Data are expressed as the mean ± SD. ^∗∗^*p* < 0.01; ^∗∗∗^*p* < 0.001.

In the previous experiments, flow cytometry analysis detected that the proliferation of MDSCs was significantly reduced after ZQT treatment. We used IF to label Ki67 (a marker for cell proliferation) in the sorted MDSCs. The results showed that the expression of Ki67 in Z-MDSCs was significantly less than C-MDSCs, which is in line with the previous finding (Figures 5(a)–5(c)). It further showed that the elimination of MDSCs was indeed remarkably augmented after ZQT administration. Besides, we found that although ZQT can affect the proliferation of MDSCs, it has no effect on the cell cycle of these cells (Figure S5C–S5E). MDSCs are bone marrow-derived cells. In order to find out whether the MDSCs inhibitory effect of ZQT was because ZQT could directly suppress the generation of MDSCs in the bone marrow background, leading to the decrease in the number of MDSCs, we carried out flow cytometry analysis. We found that there was no overt alteration in the proportion of MDSCs as well as T cells in bone marrow (Figure S4). Therefore, we ruled out the possibility that ZQT has an inhibitory effect on the generation of MDSCs. Moreover, accumulating studies have shown that Chinese medicine is frequently involved in cellular apoptosis in TME [23–25]. Therefore, on the basis of the above data, we hypothesized that ZQT triggered the apoptosis of MDSCs, resulting in a decrease in the number of these cells. In order to verify our hypothesis, we used Annexin V^+^/PI^+^ double staining and TUNEL assay to detect the apoptosis of MDSCs. Indeed, flow cytometry data revealed that the proportion of apoptotic MDSCs (Annexin V^+^ and PI^+^) was remarkably elevated in the tumors of ZQT-treated mice compared with that of saline control group (Figures 5(d)–5(e)). Moreover, TUNEL analysis further supported the above results, indicating that ZQT actually induced apoptosis of MDSCs (Figures 5(f)–5(g)). In contrast, apoptotic cells were not detected in CD3^+^ T cells (Figures S5A–S5B). Thus, the data suggest that ZQT selectively promotes massive apoptosis of MDSCs in the TME, but protects T lymphocytes from cellular apoptosis in the tumor sites. Overall, ZQT reduces the number of tumor-infiltrating MDSCs by inducing apoptosis in these cells.

### 3.6. ZQT Selectively Induced STAT3/S100A9/Bcl-2/Caspase-3 Signaling Pathway-Mediated Apoptosis in G-MDSCs

**Figure 6:**
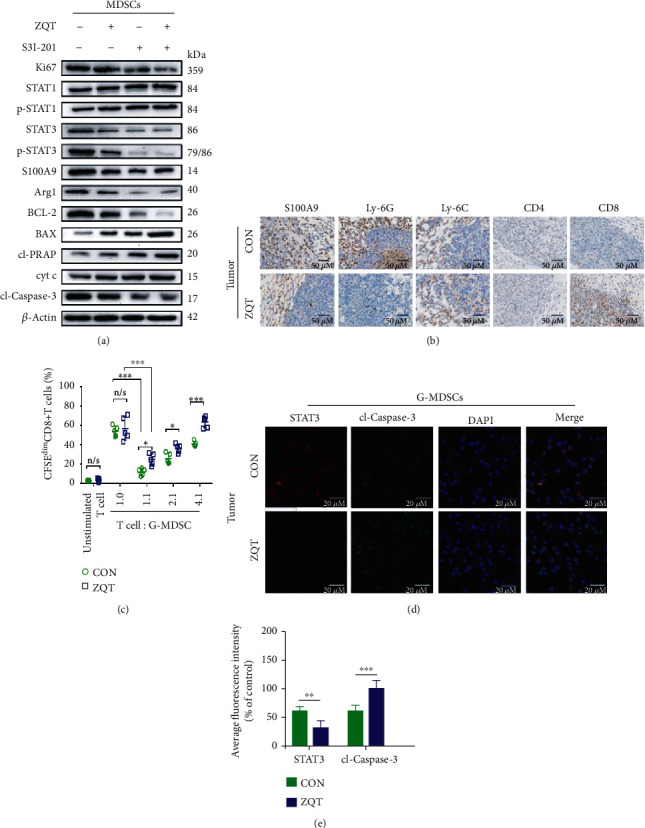
ZQT selectively induced G-MDSC apoptosis by activating the STAT3/S100A9/Bcl-2/caspase-3 signaling pathway. (a) MDSCs from the tumor tissue of tumor-bearing mice treated with normal saline, ZQT, STAT3 inhibitor (S3I-201), or ZQT combined with S3I-201 were isolated using the MojoSort Mouse Isolation Kit. The protein expression of Ki67, STAT1, p-STAT1, STAT3, p-STAT3, S100A9, Arg-1, Bcl-2, Bax, cl-PARP, cyt c, and cl-caspase-3 in MDSCs from both normal saline and ZQT-treated mice was confirmed by Western blotting. (b) Lung tumor tissues from normal saline or ZQT-treated tumor-bearing mice were stained using S100A9, Ly6G, Ly6C, CD4, and CD8 antibodies for IHC assay (scale bar: 50 *μ*M). (c) T-lymphocyte proliferation assay was used to show dose-dependent suppression of T cell proliferation by G-MDSCs isolated from tumor tissue of tumor-bearing mice treated with normal saline or ZQT. CD3^+^ T cells isolated from tumor stained with 5 *μ*M CFSE were incubated with G-MDSCs for 72 h. Meanwhile, CD3 and CD28 antibodies were added for CD3^+^ T cell stimulation. Flow cytometry was used to quantify 72 h CFSE dilution. (d, e) G-MDSCs from tumor tissue of tumor-bearing mice treated with normal saline or ZQT were isolated using flow cytometry. The purity (CD11b^+^ Ly6G^+^) was >90% as assessed, and the protein expression of STAT3 and cl-caspase-3 was determined by IF (scale bar: 20 *μ*M). *n* = 5. Data are expressed as the mean ± SD. n/s: nonstatistical significance. ^∗^*p* < 0.05; ^∗∗^*p* < 0.01; ^∗∗∗^*p* < 0.001.

According to the above data, due to the ability of ZQT to induce apoptosis in MDSCs, the infiltration of MDSCs, among which G-MDSCs was dominated, was significantly decreased, as is demonstrated by flow cytometry analysis data (Figure 3). Moreover, the expression of Arg1 and ROS that are tightly associated with the immunosuppressive capability of G-MDSCs was strikingly downregulated (Figures 4(e) and 4(f)). Therefore, we hypothesized that immunosuppressive activity of G-MDSCs was inhibited by ZQT, which subsequently contributed to apoptosis in these cells. Previous findings show that STAT3 and p-STAT3 are mainly involved in the immunosuppressive function of G-MDSCs, while STAT1 and p-STAT1 are primarily related to the immunosuppressive activity of M-MDSCs [26]. Hence, we detected STAT3/p-STAT3, STAT1/p-STAT1, and related proteins in their downstream Bcl-2/caspase-3 signaling pathway that are closely associated with apoptosis. The Western blotting results indicated that the expression of STAT3 and p-STAT3 (Ser727) was decreased in MDSCs that were isolated from tumor sites after ZQT gavage (Figure 6(a)). In addition, the antiapoptotic protein Bcl-2 was significantly decreased, while the proapoptotic protein Bax was remarkably increased in MDSCs. Caspase-3 is the most important terminal cleavage enzyme in the process of apoptosis, and its main substrate PARP (poly(ADP-ribose) polymerase) is not significantly changed [27]. However, both cleaved-caspase-3 (cl-caspase-3) and cleaved-PARP (cl-PARP) were significantly upregulated, indicating that apoptosis was triggered (Figure 6(a)). Besides, the activation of caspase-3 depends on the release of cyt-c (Cytochrome C). According to our data, the result of cyt-c was also consistent with the expression of cl-caspase-3 and cl-PARP (Figure 6(a)). Moreover, the content of Ki67 was significantly diminished, and the titer of S100A9 and Arg1, which is closely related to G-MDSCs, also showed a corresponding decrease, indicating that G-MDSCs activity was decreased and apoptosis occurred after ZQT treatment (Figure 6(a)). Based on the above results, STAT3 plays a dominant role in ZQT's antitumor activity. To further verify the role of STAT3 in the ZQT-induced antitumor effect, we applied the STAT3 inhibitor (S3I-201) at 5 mg/mL, 3 times a week in orthotopic lung cancer mice via intraperitoneal injection for 4 weeks. After the treatment of S3I-201, ZQT, and S3I-201+ZQT, the results showed that the survival-prolonging effect of S3I-201 was comparable to that of the ZQT and S3I-201+ZQT groups, with no significant difference among these three groups (Figure S7A). The administration of S3I-201, combined with ZQT or not, significantly downregulated Ki67, S100A9, Bcl-2, and Arg1 protein expression, but upregulated cleaved-PARP, cyt-c, and cleaved-caspase-3 protein expression as expected, indicating the increased apoptosis and decreased population of G-MDSCs (Figures S7B–S7C). These data were also consistent with the survival results. Besides, ROS is a common marker of immunosuppressive activity of G-MDSCs and is also closely related to the activation of STAT3. Hence, we detected the titer of ROS post usage of S3I-201, ZQT, and S3I-201+ZQT. We found that all of these three treatments could remarkably reduce the content of ROS compared with the control group, and there was no significant difference among these three treatments (Figure S7D). Taken together, ZQT induced apoptosis in G-MDSCs through the STAT3/S100A9/Bcl-2/caspase-3 signaling pathway-mediated inhibition of the immunosuppressive activity of G-MDSCs, exerting antitumor effect.

### 3.7. The Tumor Inhibitory Effect of ZQT Depended on the Inhibition of G-MDSCs

**Figure 7:**
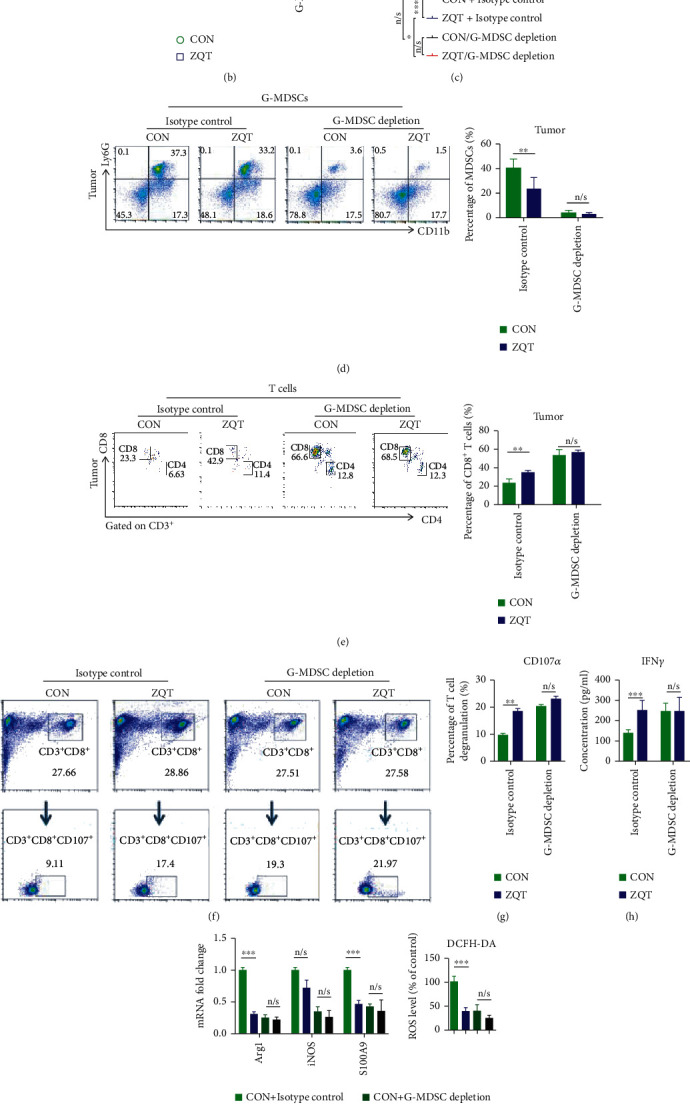
ZQT induced apoptosis in G-MDSCs in TME and inhibited its immunosuppressive activity by activating the STAT3/S100A9/Bcl-2/caspase-3 signaling pathway. Mice were treated with anti-Ly6G neutralizing antibody or isotype control by intraperitoneal injection for 14 days before surgery, after which model mice were given maintenance treatment every other day. (a, b) The bioluminescence images of model mice on day 7 and day 28 after surgery. (c) Survival curves. (d, e) The percentage of G-MDSCs, CD3^+^ T cells, CD4^+^ T cells, and CD8^+^ T cells in tumor tissue were analyzed by flow cytometry. (f, g) CD3^+^ T cells isolated from the tumor tissue of tumor-bearing C57BL/6 mice in different groups were cocultured with LLC cells for 4 h before they were incubated with CD3, CD8, and CD107*α* antibodies. Events shown were finally gated on CD3 and CD8. (h) ELISA analysis for the expression of IFN-*γ* in cells from tumor tissue. (i) RT-qPCR analysis of Arg-1, iNOS, and S100A9 gene expression and flow cytometry for ROS production. (j) IHC staining for Ly6G and CD8 in tumor tissue (scale bar: 50 *μ*M). *n* = 5. Data are expressed as the mean ± SD. n/s: nonstatistical significance. ^∗^*p* < 0.05; ^∗∗^*p* < 0.01; ^∗∗∗^*p* < 0.001.

To further illustrate whether G-MDSCs play a crucial role in ZQT-mediated tumor suppressive effect, we injected the Ly6G monoclonal antibody into mice to deplete G-MDSCs. Model mice were randomly divided into four groups: the isotype saline control group, the isotype ZQT control group, the G-MDSC depletion saline control group, and the G-MDSC depletion ZQT group (Figure 7(a)). The Ly6G antibody was intraperitoneally injected into the mice twice a week at 10 mg/kg each time, starting from two weeks before the intrapulmonary surgery, until the end of the entire experimental period. The flow cytometry data showed that the depletion efficiency of G-MDSCs can reach over 90% (Figure S6A). Next, we performed in vivo imaging to detect the inhibitory effect of ZQT on tumor growth in mice after G-MDSC exhaustion. The results showed that when G-MDSCs were depleted, the antitumor activity of ZQT, which was indicated by the inhibition of the luminescence signal in the lung tumor, was nearly the same as that of the G-MDSC depletion saline control group, and its survival-prolonging effect was also comparable to that of the control group (Figures 7(a)–7(c)). Findings from flow cytometry analysis confirmed that the number of G-MDSCs was dramatically decreased in the G-MDSC depletion groups compared with the isotype control group. When G-MDSCs were absent, the T cell proliferation-promoting activity and the cytotoxicity of CD8^+^ T cells in the ZQT group were almost the same as those of the control group, which are in agreement with the imaging and survival data (Figures 7(d)–7(h) and Figures S6B–S6G). After G-MDSC depletion, the RT-qPCR results showed that the downregulation effect of ZQT on the immunosuppressive markers of MDSCs (Arg1, iNOS, and S100A9) showed no significant difference compared with the control group (Figure 7(i)). What's more, the expression level of the markers of G-MDSCs (Ly6G) and CD8^+^ T cells (CD8) also had no overt changes compared with that of the control group (Figure 7(j)). In general, when G-MDSCs were depleted by the Ly6G monoclonal antibody, the tumor suppressive effect of ZQT disappeared, suggesting that ZQT exerted its tumor inhibitory function mainly via reducing the G-MDSC subtype population and inhibiting its immunosuppressive activity.

## 4. Discussion

In this study, the orthotopic lung cancer model was selected for two main reasons. The first reason is based on the basic theory of TCM, which states that the principle of TCM treatment is “according to the etiology of the disease, using the corresponding drugs.” This means that “a disease is caused by a certain reason, and therefore using a certain drug that targets the cause of the disease to treat the disease.” In animal studies, subcutaneous inoculation of tumor cells in mice is the most common and convenient method to establish a tumor model. However, the TME of subcutaneous tumors and their surrounding TME are different from that of orthotopic lung tumors. Extensive researches showed that the orthotopic lung tumor nodules are more likely to metastasize than subcutaneous ones, indicating that the orthotopic tumors are more relevant to the clinical situation. In addition, literatures have shown that the false positive rate of drug efficacy in subcutaneous tumors is much higher than that in orthotopic tumors [28, 29]. Another reason for using the orthotopic lung tumor model rather than other tumor models is that the therapeutic principles of “drug meridian tropism” also play a vital role in TCM theory. Channel tropism refers to the therapeutic action of drugs on a certain part of the human body, or in other words, the target of the medicine in the human body. There are nine commonly used Chinese herbs in ZQT (Ze Qi, Zhi Ban Xia, Zi Shen, Bai Qian, Sheng Jiang, Gui Zhi, Huang Qin, Ren Sheng, and Gan Cao). These herbs are combined according to the compatibility principle of emperor, minister, adjuvant, and courier (Jun, Chen, Zuo, and Shi) to create a synergistic therapeutic effect. Moreover, the tropism of these nine herbs belongs to the lung meridian, which means that the action site of ZQT is the lung.

TME provides a site where cancer cells interact with stromal cells to promote cancer progression. Numerous literatures have shown that TCM is capable of modulating TME [30–32]. Consistent with previous research, in the present study, we found that ZQT could significantly inhibit tumor development by reducing the MDSC population and inhibiting their immunosuppressive activity to restore the bioactivity of T cells in TME. MDSCs are a heterogeneous population of immature cells in the immune system, and they come to the forefront as stromal cells that orchestrate the immunosuppressive TME [33]. A hallmark of MDSCs is that they efficiently suppress effector T cell responses, including T cell proliferation and functional properties. In most cancers, G-MDSCs are predominant, representing approximately three-fourths of all MDSCs. The major factors involved in G-MDSC-mediated immunosuppression mainly include Arg1 and ROS, as well as others [15]. In our previous study, we have revealed that the TCM formula ZQT played a direct role in tumor inhibition by inducing intrinsic apoptosis and blocking the cell cycle in a subcutaneous lung cancer model established in immune-deficient mice. However, more and more evidence show that TCM is capable of regulating TME [7, 8]. Therefore, we explored whether ZQT had a significant regulatory effect on TME of the orthotopic lung cancer mouse model established with immune competent mice. To the best of our knowledge, our study is the first in-depth study to investigate the modulatory effect of ZQT on the G-MDSC subgroup of MDSCs in the TME. In our study, we found that the population of MDSCs (mainly G-MDSCs) was obviously decreased after ZQT therapy due to ZQT-induced apoptosis in G-MDSCs via accumulation of cl-PARP and cl-caspase-3 through diminishing antiapoptotic protein Bcl-2 and enhancing proapoptotic protein Bax. Meanwhile, the immunosuppressive activity of MDSCs, including the expression of Arg-1, was also diminished by ZQT, and the infiltration of CD8^+^ T cells as well as its cytotoxicity activity was elevated. Numerous researches indicate that Arg-1 is mostly expressed by G-MDSCs following the activation of STAT3, leading to the growth arrest of antigen-activated T cells, while M-MDSCs mostly express iNOS through the activation of STAT1. In our study, we found that after ZQT treatment, the protein levels of p-STAT3, Arg-1, and S100A9 that are tightly associated with G-MDSCs were significantly downregulated, while the content of CD107*α* and IFN-*γ* of CD8^+^ T cells was markedly upregulated. These results indicate that the tumor inhibition effect of ZQT is mainly mediated by triggering G-MDSC apoptosis via the STAT3/Bcl-2/caspase-3 signaling pathway to restore effective immune activity of cytotoxic T cells. Moreover, when G-MDSCs were exhausted by the Ly6G monoclonal antibody, the antitumor activities of ZQT, including weakening the immunosuppressive effect of G-MDSCs on T cells, inhibiting pulmonary luminescence signals of tumor-bearing mice, and extending their survival, were depleted (Figure 7(a)). These data further confirmed that ZQT-mediated tumor suppression is achieved through the inhibition of G-MDSCs via attenuating their immunosuppressive activity and inducing apoptosis in these cells.

**Figure 8:**
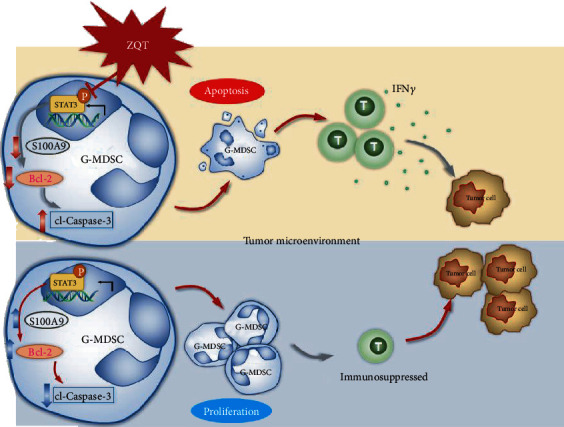
The immune regulatory activity of TCM formula ZQT in TME. ZQT induces the apoptosis of G-MDSCs via the STAT3/S100A9/Bcl-2/caspase-3 signaling pathway, resulting in a significant decrease in G-MDSCs and their immunosuppressive activity, consequently promoting the infiltration and killing activity of CD8^+^ T cells, leading to the inhibition of the proliferation of tumor cells.

In our previous studies, ZQT could induce apoptosis in human H460 and A549 lung cancer cells through activating the p53 signaling pathway in immunodeficient nude mice. In this study, we revealed that ZQT played an immunomodulatory role in an orthotopic lung cancer mouse model via reducing the population of G-MDSCs and inhibiting their immunosuppressive activity in TME by activating the STAT3/Bcl-2/caspase-3 signaling pathway. The above findings indicate that ZQT possesses multitarget antitumor activity, which is in line with the clinical application of ZQT (Figure 8). Our team is exploring and illustrating the multiple tumor-inhibiting effects of classic TCM formulas, such as ZQT and Yu-Ping-Feng (YPF) ([19, 31]).

Unlike western medicine, TCM has been used in clinical practice for thousands of years in the form of formulas (mixture/water extract). One of the major limitations of a TCM formula is the complexity of its components that restricts in-depth research on these medicines. In this study, we explored the effect of the ZQT formula, which consists of nine different Chinese herbs, on orthotopic lung tumor-bearing mice. Although the ZQT formula showed potent antitumor activity, we cannot determine which components of the formula mediated this antitumor effect. If the effective components are not clear, it is difficult to explore the mechanism of the formula in depth. Thus, in the follow-up study, we will try to identify the effective main antitumor components in the ZQT formula to further investigate its tumor inhibitory activity in lung cancer and reveal the underlying mechanism. In addition, with the modernization of TCM, more and more experts who are experienced in the combined therapy of traditional Chinese medicine and western medicine advocate combining the effective components of every single herb from TCM formulas as a new compound to replace the previous TCM formula. However, there are many difficulties in practice. For example, each herb in the formula may contain multiple active components, which makes it difficult to find out all the effective components that contribute to the therapeutic efficacy of a formula that often includes several herbs. Moreover, their pharmacokinetics/pharmacodynamics in the body either as an active component compound or as a TCM formula may also be different to illustrate. Therefore, more efforts are needed to explain the clinical effectiveness of TCM.

## 5. Conclusions

Taken together, this study is the first to reveal the immune regulatory activity of the TCM formula ZQT in TME. We demonstrated that ZQT oral administration significantly inhibited the tumor growth in the lung and prolonged the survival of the orthotopic lung tumor-bearing mice. The underlying mechanism may be that ZQT induces the apoptosis of G-MDSCs via the STAT3/S100A9/Bcl-2/caspase-3 signaling pathway (Figure 8). Therefore, the population of G-MDSCs and its immunosuppressive activity were remarkably reduced, consequently promoting the infiltration and killing activity of CD8^+^ T cells.

## Data Availability

The data used to support the findings of this study are available from the corresponding authors upon request.

## Abbreviations

ZQT: Ze-Qi-Tang
NSCLC: Non-small-cell lung cancer
MDSCs: Myeloid-derived suppressor cells
G-MDSCs: Granulocytic myeloid-derived suppressor cells
M-MDSCs: Monocytic myeloid-derived suppressor cells
TME: Tumor microenvironment
PI: Propidium iodide
IF: Immunofluorescence
IHC: Immunohistochemistry
TUNEL: Terminal deoxynucleotidyl transferase (TdT) dUTP nick-end labeling
Z-MDSCs: MDSCs from ZQT-treated tumor-bearing mice
C-MDSCs: MDSCs from control-treated tumor-bearing mice
IFN-*γ*: Interferon-*γ*
Arg-1: Arginase 1
iNOS: Inducible nitric oxide synthase
ROS: Reactive oxygen species
S100A9: S100 calcium-binding protein A9
STAT: Signal transducer and activator of transcription
Caspase-3: Cysteine aspartate-specific protease 3
Bcl-2: B-cell lymphoma 2
cl-PARP: Cleaved-poly(ADP-ribose) polymerase
Cyt c: Cytochrome c
YPF: Yu-Ping-Feng.

## Supplementary Materials

Supplementary Materials Supplementary Table 1: main composition of ZQT. Supplementary Figure 1: schematic diagram of constructing an orthotopic lung cancer mouse model. Supplementary Figure 2: effect of ZQT on immune cells closely related to TME remodeling. Supplementary Figure 3: ZQT reduced the level of MDSCs and increased the number of CD8^+^ T cells in the spleen, but did not influence the MDSCs and T cells in bone marrow. Supplementary Figure 4: ZQT has no significant effect on MDSCs and T cells in bone marrow. Supplementary Figure 5: ZQT protects T lymphocytes from cellular apoptosis in the tumor sites. Supplementary Figure 6: knockout efficiency of G-MDSCs and ZQT-induced apoptosis of G-MDSCs in spleen. Supplementary Figure 7: effect of STAT3 inhibitor S3I-201 on G-MDSCs.
