# Supplementary material for: Ze-Qi-Tang Formula Induces Granulocytic Myeloid-Derived Suppressor Cell Apoptosis via STAT3/S100A9/Bcl-2/Caspase-3 Signaling to Prolong the Survival of Mice with Orthotopic Lung Cancer
Source: Mediators Inflamm. 2021 Apr 1;2021:8856326. doi: 10.1155/2021/8856326 (PMC8035014; doi:10.1155/2021/8856326)
Supplement: Supplementary Materials — Supplementary Table 1: main composition of ZQT. Supplementary Figure 1: schematic diagram of constructing an orthotopic lung cancer mouse model. Supplementary Figure 2: effect of ZQT on immune cells closely related to TME remodeling. Supplementary Figure 3: ZQT reduced the level of MDSCs and increased the number of CD8+ T cells in the spleen, but did not influence the MDSCs and T cells in bone marrow. Supplementary Figure 4: ZQT has no significant effect on MDSCs and T cells in bone marrow. Supplementary Figure 5: ZQT protects T lymphocytes from cellular apoptosis in the tumor sites. Supplementary Figure 6: knockout efficiency of G-MDSCs and ZQT-induced apoptosis of G-MDSCs in spleen. Supplementary Figure 7: effect of STAT3 inhibitor S3I-201 on G-MDSCs. [file 8856326.f1.docx]

**Supplementary material**

**Supplementary table 1**

Main composition of ZQT

| **Main composition** | **Latin scientific name** | **Parts used** | **Amount (g)** |
| --- | --- | --- | --- |
| Euphorbia helioscopia (Ze Qi) | *Euphorbia helioscopiaL.* | Herba | 30 |
| Processed Pinellia ternata (Zhi Ban Xia) | *Pinellia ternata (Thunb.) Breit.* | Tuber | 15 |
| Salvia chinensis (Zi Shen) | *Rubia yunnanensis Diels* | Herba | 10 |
| Cynanchum glaucescens (Bai Qian) | *Cynanchum glaucescens (Decne.) Hand.-Mazz.* | Rhizome | 10 |
| Fresh ginger (Sheng Jiang) | *Zingiber officinale Roscoe* | Rhizome | 10 |
| Cassia twig (Gui Zhi) | *Cinnamomum cassia Presl* | Twig | 6 |
| Scutellaria baicalensis(Huang Qin) | *Scutellaria baicalensis Georgi* | Radix | 6 |
| Ginseng (Ren Sheng) | *Panax ginseng C. A. Mey.* | Radix | 6 |
| Liquorice(Gan Cao) | *Glycyrrhiza uralensis Fisch.* | Rhizoma | 6 |


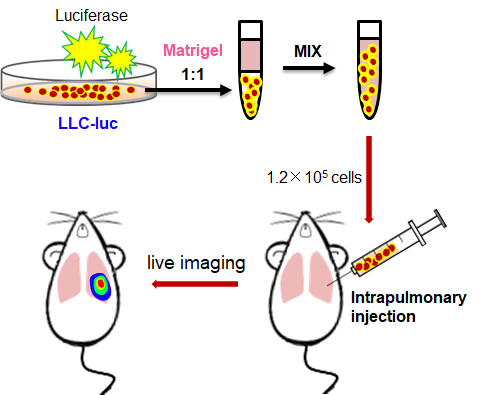


**Supplementary Figure 1.** **Schematic diagram of constructing an orthotopic lung cancer mouse model.**


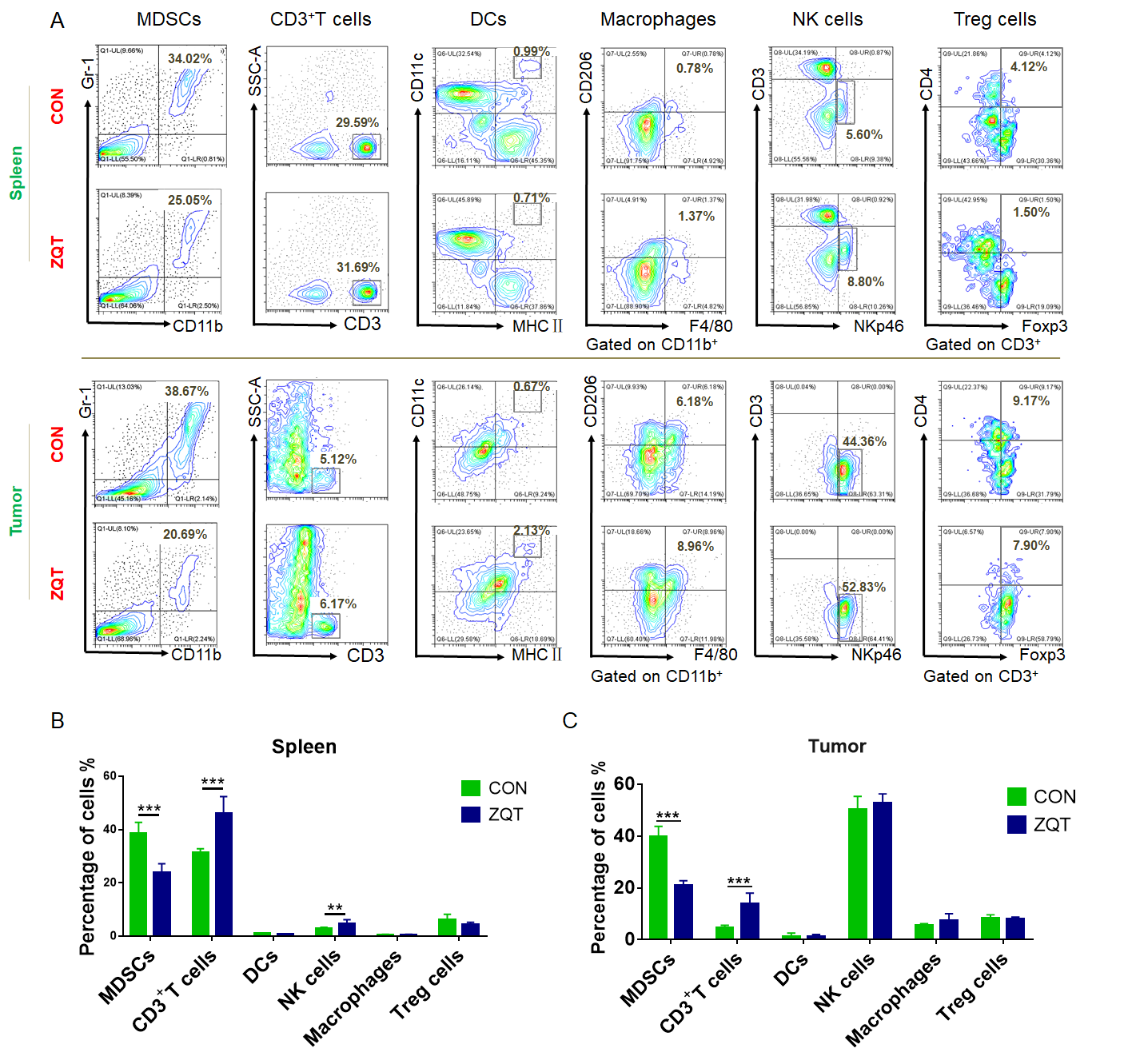


**Supplementary Figure 2.** **Effect of ZQT on immune cells closely related to TME remodeling.** Cells from the spleen and tumor tissue of tumor bearing mice treated with normal saline or ZQT for 28 days were analyzed by flow cytometry after stained with CD11b, Gr-1, CD3, MHC Ⅱ, CD206, F4/80, NKp46, CD4 and Foxp3. (A-C) Percentages of MDSCs, CD3^+^ T cells, DCs, NK cells, macrophage and Treg cells in spleen or tumor. n=5. Data are mean ± SD, **, p<0.01; ***, p<0.001.


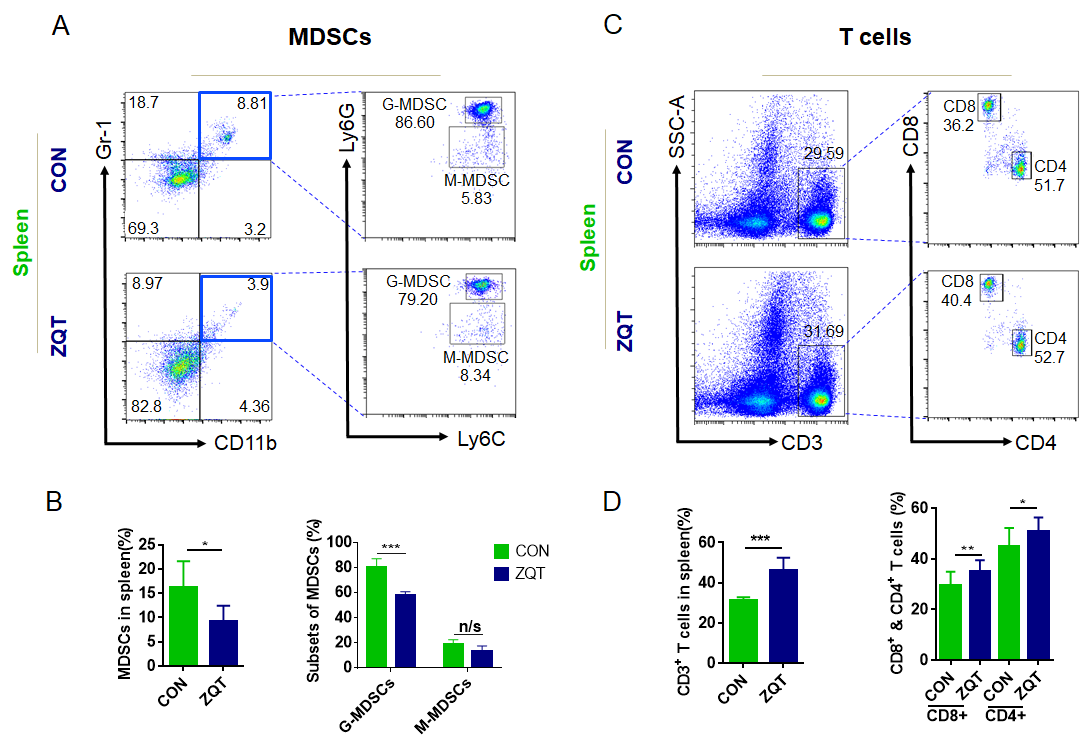


**Supplementary Figure 3.** **ZQT reduced the level of MDSCs and increases the number of CD8 ^+^ T cells in the spleen, but did not influence the MDSCs and T cells in the bone marrow.** Cells from spleen or bone marrow of tumor bearing mice treated with normal saline or ZQT for 28 days were analyzed by flow cytometry after stained with CD11b, Gr-1, Ly6G, Ly6C, CD3, CD4 and CD8. (A-D) Percentages of MDSCs, G-MDSCs, M-MDSCs, CD3^+^ T cells, CD4^+^ T cells and CD8^+^ T cells in spleen. (E-F) Percentages of MDSCs, CD4^+^ T cells and CD8^+^ T cells in spleen. n=5. Data are mean ± SD, n/s, non-statistical significance; *, p<0.05; ***, p<0.001.


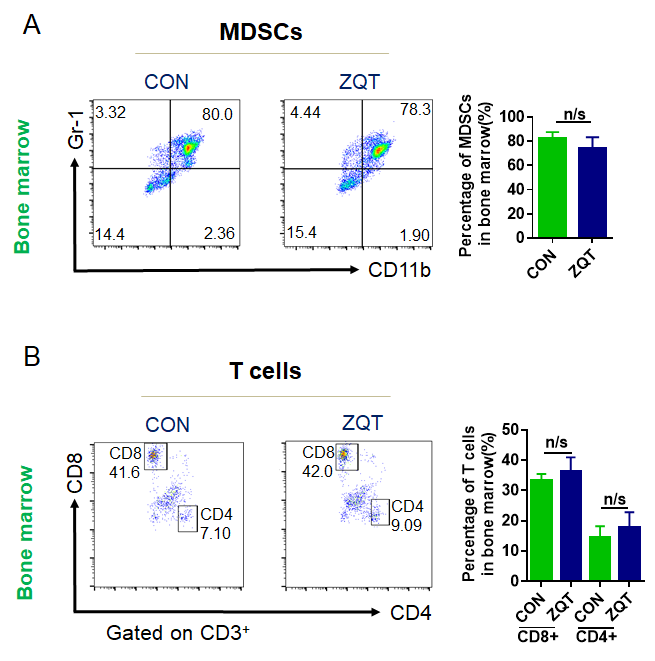


**Supplementary Figure 4. ZQT has no significant effect on MDSCs and T cells in bone marrow.** Cells from bone marrow of tumor bearing mice treated with normal saline or ZQT for 28 days were analyzed by flow cytometry after stained with CD11b, Gr-1, Ly6G, Ly6C, CD3, CD4 and CD8. (A-B) Percentages of MDSCs, CD4^+^ T cells and CD8^+^ T cells in bone marrow. n=5. Data are mean ± SD. n/s, non-statistical significance.


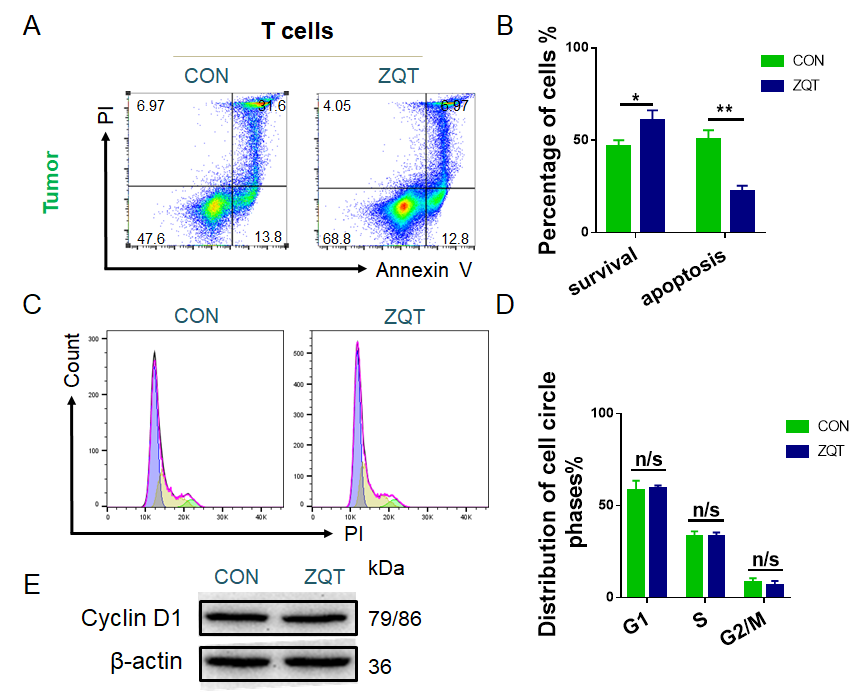


**Supplementary Figure 5. ZQT protects T lymphocytes from cellular apoptosis in the tumor sites.** (A-B) CD3^+^ T cells isolated from tumor tissue of normal saline or ZQT-treated mice were stained with Annexin Ⅴ and PI. Percentage of surviving and apoptosis cells were analyzed by flow cytometry. (C-E) MDSCs isolated from tumor tissue of normal saline or ZQT-treated mice were isolated using the MojoSort Mouse Isolation Kit. The cell circle phases were analyzed by flow cytometry. The protein expression of cyclin D1 was confirmed by Western blotting. n=5. Data are mean ± SD, *, p<0.05; **, p<0.01.


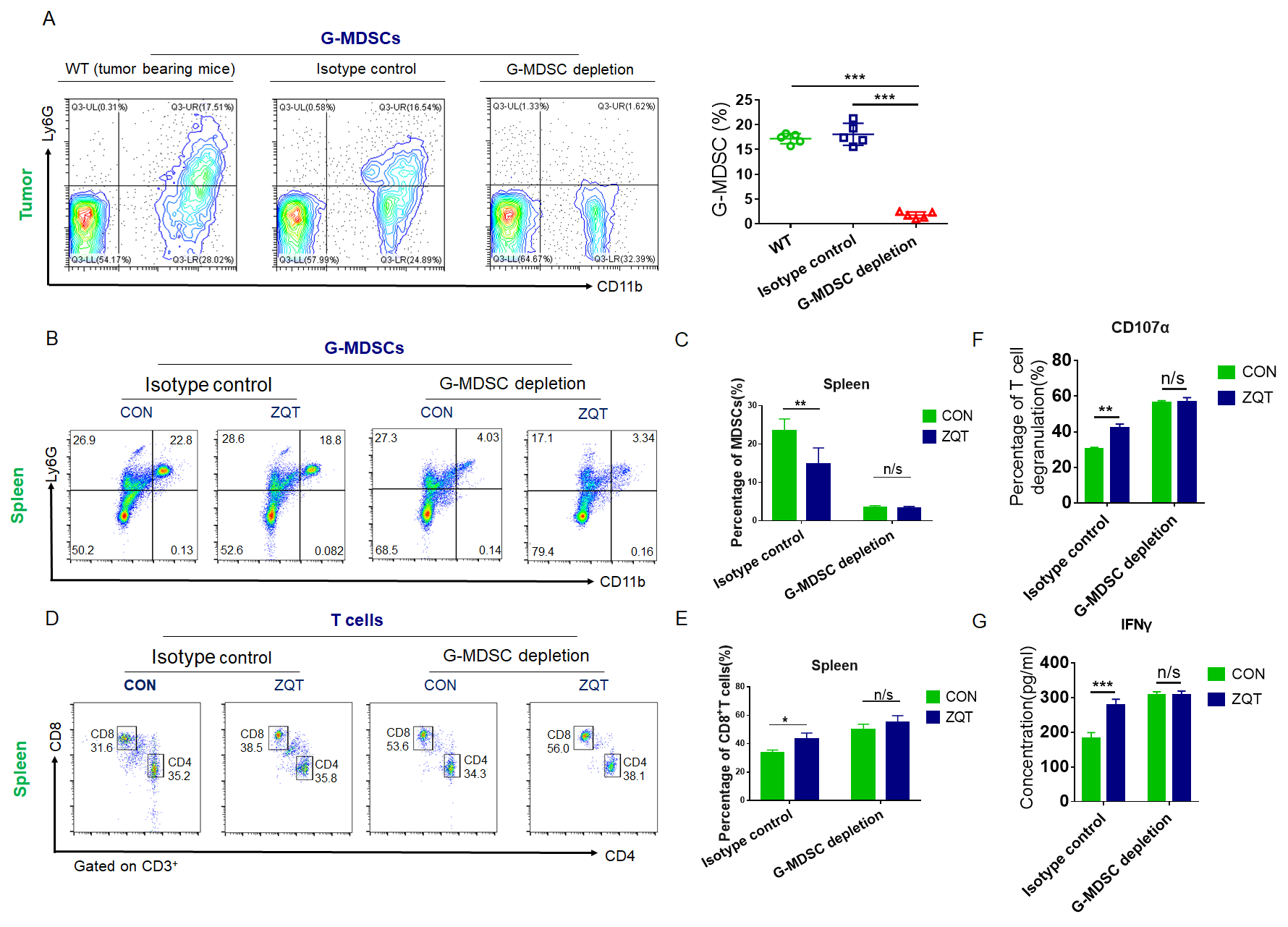


**Supplementary Figure 6.** **Knockout efficiency of G-MDSCs and ZQT-induced apoptosis of G-MDSCs in spleen.**  Mice were treated with anti-Ly6G neutralizing antibody or isotype control by intra-peritoneal injections for 14 days before surgery, after which model mice were given maintenance treatment every other day. (A) The percentage of G-MDSCs (CD11b^+^ Ly6G^+^) isolated from tumor tissue in WT, G-MDSCs depletion or isotype control mice. (B-E) The percentages of splenic G-MDSCs, CD4^+^ T cells and CD8^+^ T cells in all groups were analyzed by flow cytometry. (F) Cells from tumor tissue were incubated with CD3, CD8, CD107α for 4 h and analyzed by flow cytometry. (G) ELISA analysis for the expression of IFNγ in the serum of tumor bearing mice in all groups. n=5. Data are mean ± SD, n/s, non-statistical significance; *, p<0.05; **, p<0.01; ***, p<0.001.


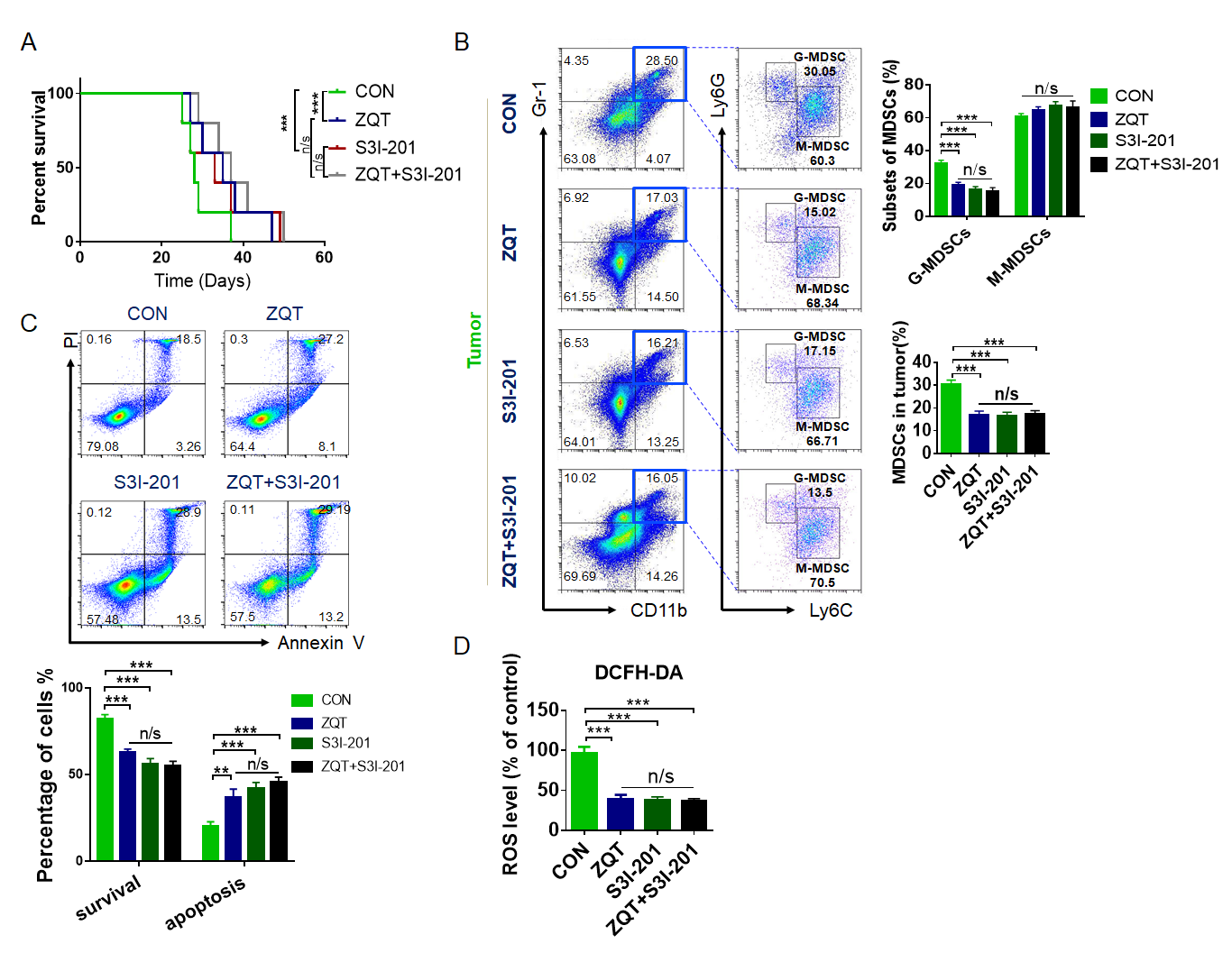


**Supplementary Figure 7. Effect of STAT3 inhibitor S3I-201 on G-MDSCs.** Mice were intragastrically treated with daily dose of 200 μl Ze-Qi-Tang, intraperitoneally injected with 5 mg/ml STAT3 inhibitor (S3I-201), separately or combined, 3 times a week for 4 weeks. The normal saline was used as control. (A) Survival curves. (B) The percentages of tumor G-MDSCs in all groups were analyzed by flow cytometry. (C) MDSCs isolated from tumor tissue of all groups were stained with Annexin Ⅴ and PI. Percentage of surviving and apoptosis cells were analyzed by flow cytometry. (D) ROS assay for ROS production in G-MDSCs. **, p<0.01; ***, p<0.001.
